# A Feedback Mechanism to Control Apoptosis Occurs in the Digestive Gland of the Oyster *Crassostrea gigas* Exposed to the Paralytic Shellfish Toxins Producer *Alexandrium catenella*

**DOI:** 10.3390/md12095035

**Published:** 2014-09-25

**Authors:** Jean-Luc Rolland, Walid Medhioub, Agnes Vergnes, Celina Abi-khalil, Véronique Savar, Eric Abadie, Estelle Masseret, Zouher Amzil, Mohamed Laabir

**Affiliations:** 1 IFREMER, Université Montpellier 2, Centre National de la Recherche Scientifique, IRD, UM1, UMR 5119 “Ecologie des Systèmes Marins Côtiers”, Place E. Bataillon, CC93, Montpellier cedex 5 34095, France; E-Mails: agnes.vergnes@ifremer.fr (A.V.); celina.abikhalil@univ-montp2.fr (C.A.); estelle.masseret@univ-montp2.fr (E.M.); mohamed.laabir@univ-montp2.fr (M.L.); 2 INSTM, Institut National des Sciences et Technologies de la Mer, Laboratoire d’Aquaculture, 28 rue du 2 mars 1934, Salammbô 2025, Tunisia; E-Mail: medhwalid@yahoo.fr; 3 IFREMER, Laboratoire Phycotoxines (PHYC), Rue de l’Ile d’Yeu BP 21105, Nantes Cedex 3 44311, France; E-Mails: veronique.savar@ifremer.fr (V.S.); zouher.amzil@ifremer.fr (Z.A.); 4 IFREMER, Département des laboratoires côtiers Environnement littoral et Ressources aquacoles (LER-LR), Avenue Jean Monnet, BP 171, Sète Cedex 34203, France; E-Mail: eric.abadie@ifremer.fr

**Keywords:** shellfish, toxin, biomarker, expression, phytoplankton

## Abstract

To better understand the effect of Paralytic Shellfish Toxins (PSTs) accumulation in the digestive gland of the Pacific oyster, *Crassostrea gigas*, we experimentally exposed individual oysters for 48 h to a PSTs producer, the dinoflagellate *Alexandrium catenella*. In comparison to the effect of the non-toxic *Alexandrium tamarense*, on the eight apoptotic related genes tested, Bax and BI.1 were significantly upregulated in oysters exposed 48 h to *A. catenella*. Among the five detoxification related genes tested, the expression of cytochrome P450 (CYP1A) was shown to be correlated with toxin concentration in the digestive gland of oysters exposed to the toxic dinoflagellate. Beside this, we observed a significant increase in ROS production, a decrease in caspase-3/7 activity and normal percentage of apoptotic cells in this tissue. Taken together, these results suggest a feedback mechanism, which may occur in the digestive gland where BI.1 could play a key role in preventing the induction of apoptosis by PSTs. Moreover, the expression of CYP1A, Bax and BI.1 were found to be significantly correlated to the occurrence of natural toxic events, suggesting that the expression of these genes together could be used as biomarker to assess the biological responses of oysters to stress caused by PSTs.

## 1. Introduction

The Pacific oyster *Crassostreas gigas*, like many other bivalve mollusks, is a filter feeder that consumes microphytoplankton, including toxic dinoflagellates when they occurred. Paralytic Shellfish Toxins (PSTs) are naturally produced by some cyanobacteria and a number of toxic dinoflagellate species including *Alexandrium catenella*, *Alexandrium minutum*, *Alexandrium tamarense*, *Pyrodinium bahamense* and *Gymnodinium catenatum* [1,2,3]. Saxitoxin and its congeners can be divided into three categories: the carbamate compounds, which include Saxitoxin, neo-Saxitoxin and Gonyautoxins 1–4; the *N*-sulfocarbamoyl compounds, which include the C and B toxins; and the decarbamoyl compounds. Saxitoxin is one of the most potent and deadly toxins in the world and was shown to be a highly selective blocker of Na^+^ channels in excitable cells, thereby affecting nerve impulse generation [4] and can lead, in extreme cases, to human death [5]. *In situ*, oysters feeding on toxic dinoflagellates accumulate PSTs. When concentration exceeds the sanitary threshold (800 μg saxitoxin equiv/kg wet weights) farms are closed resulting in economic losses [6]. 

Several studies have described deleterious effects of PSTs on physiological and cellular processes in oysters including filtration activity reduction [7], oxygen consumption change [8], pseudo-feces production decrease [9], metabolic rate increase [10], reproductive development anomalies [11], alteration in feeding and digestive capacity [12,13,14,15,16], and modulation of gene expression [17]. Moreover, immune cell alteration has been reported including an adhesion alteration, phagocytosis inhibition [18,19], and apoptosis [20]. 

Few data are available on the effect of PSTs on the digestive gland. PSTs can accumulate in the DG [7,21] and are able to induce modulation of the expression of key genes (α-amylase and triacylglycerol lipase B) involved in the digestive process [16]. No other effects were observed suggesting complex mechanisms occurring in this tissue. In the present study we investigate specific relationships between PSTs accumulation and metabolic process that occur on the digestive gland of *C. gigas* exposed to toxic strain of *A. catenella* in comparison with non-toxic *A. tamarense*. To do this, the temporal expressions of 5 putative related genes known to be involved in detoxification processes and eight key genes known to be involved in apoptosis were determined. These genes were previously identified from the 210,895 ESTs from *C. gigas* reported in the National Center for Biotechnology Information database of May 2013 or described by Medhioub *et al*. in 2013 [15]. We also look at caspase-3/7 activity, Reactive oxygen species production, and the appearance of apoptotic cells in digestive gland tissue.”

## 2. Results and Discussion

### 2.1. Temporal Expression of the Genes Related to Detoxification and Apoptotic Processes

On the five detoxification-related genes tested, Glutathione S-transferase (GST) transcript was over-expressed after exposure of oysters to the two tested *Alexandrium* species. Cytochrome P450 subfamily 1A (CYP1A) transcript was over-expressed when oysters were fed the toxic microalgae *Alexandrium catenella*.

**Figure 1:**
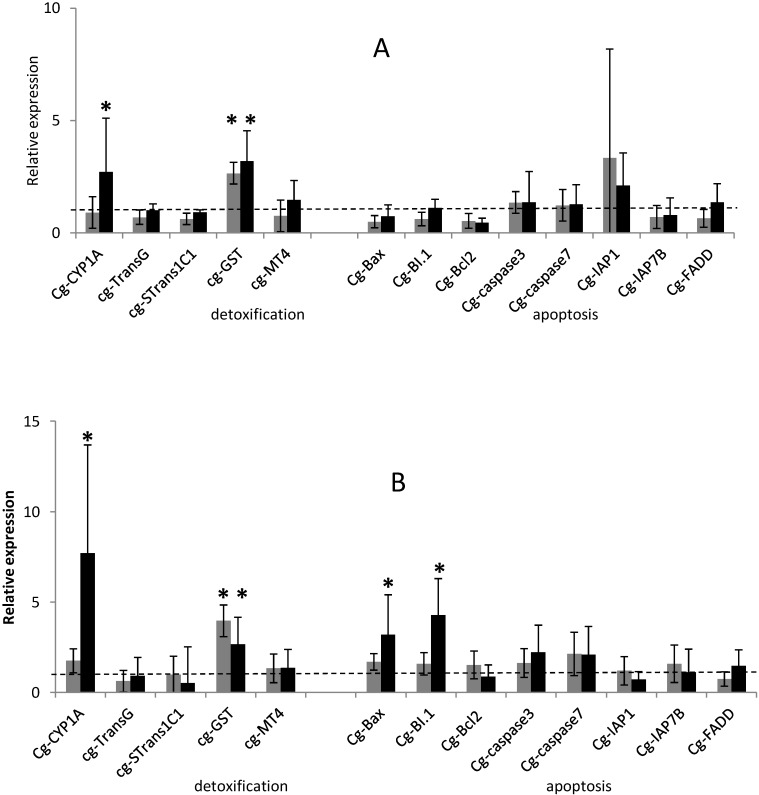
Relative expression of 5 detoxification-related gene transcripts and 8 apoptotic-related gene transcripts (normalized to ribosomal protein F40) in the digestive gland of *Crassostrea gigas* exposed for 29 h (**A**) and 48 h (**B**) to *Alexandrium tamarense* (**grey**) or to *Alexandrium catenella* (**black**) related to the non exposed oysters. The dashed line represents the mean value of the control group (non exposed oysters) for each gene. Bars represent mean ± standard errors (*N* = 10 animals). * *p <* 0.01.

GST transcripts level were significantly (*p <* 0.01) up-regulated 3.2 and 2.6-fold in the digestive gland (DG) of oysters exposed 29 h and 48 h to *A. catenella*, respectively (Figure 1). This same gene GST showed higher transcript levels 2.6 and 3.9-fold in DG of oysters exposed 29 h and 48 h to non-toxic *A. tamarense*, respectively (Figure 1). Glutathione S-transferase belongs to a large super-family of multi-functional enzymes primary involved in cellular detoxification by catalyzing the nucleophilic attack by glutathione (GSH) on electrophilic compounds with a wide range of endogenous and xenobiotic agents, including environmental toxins and oxidative stress products [22,23,24,25,26,27]. GST transcript abundance was previously demonstrated to be over expressed in oysters exposed to toxic and non toxic cells of the dinoflagellate *Gymnodinium catenatum* [17]. Similarly, in our experiment, GST transcript level was up-regulated in oysters exposed to the PSTs producer *A. catenella* but also to non toxic *A. tamarense*. These results suggest that the increase in GST transcript level is not related to PSTs accumulated in the DG but could be related to respiratory burst induced by the biotransformation and disposition of a wide range of endogenous dinoflagellates compounds [28]. 

CYP1A transcripts were significantly over-expressed (*p <* 0.01) at 29 h and 48 h (2.7- and 7-fold, respectively) in the DG of oysters exposed to *A. catenella*, in comparison to not fed oysters or those exposed to *A. tamarense* (Figure 1). The cytochrome P450 family is a large and diverse group of enzymes that generally constitute the first enzymatic defense against foreign molecules. In bivalve mollusks, studies investigated variations in CYP1A transcripts in relation to several biotic and abiotic factors [29,30]. CYP1A transcripts have been demonstrated to be up-regulated in gills of oysters exposed to changing water temperature [31] and in DG of oysters exposed to hydrocarbon contamination [32]. Unlike *CYP1A*, *C. gigas* hemocytes exposure to brevetoxin led to a decrease in transcript level of *CYP356A1* a CYP450 isoform [33]. 

On the eight apoptotic-related genes tested, *BI*.*1* and *Bax* were significantly over-expressed (*p <* 0.01) in the DG of oysters exposed 48 h to *A. catenella*, presenting an increase (4.3- and 3.1-fold, respectively) in comparison to oyster non-exposed (Figure 1B). Interestingly, transcript levels of the other *C. gigas* detoxification and apoptotic-related genes did not show any significant modulation under the different treatments after 29 h and 48 h of exposure (Figure 1). The intrinsic pathway of apoptosis which implies the cytochrome c release from mitochondria appears to be largely mediated by direct or indirect ROS action [34]. B-cell lymphoma 2 (Bcl-2) family proteins, composed of pro- and anti-apoptotic members, regulate this process by releasing apoptotic signals from the mitochondria. In our experiment, on the pro-apoptotic gene tested, *Bax* was significantly over-expressed after 48 h exposure time of oysters to *A. catenella* suggesting that the intrinsic pathway of apoptosis has been activated. In mammals, Bax protein members play a central role in the induction of the mitochondrial apoptosis pathway by promoting the release of apoptotic factors, such as cytochrome c into the cytosol, responsible for further activation of the executioner caspases (cysteine aspartate proteases) family of proteins, which plays a central role in the execution-phase of cell apoptosis [34,35,36,37,38,39].

### 2.2. ROS Production

**Figure 2:**
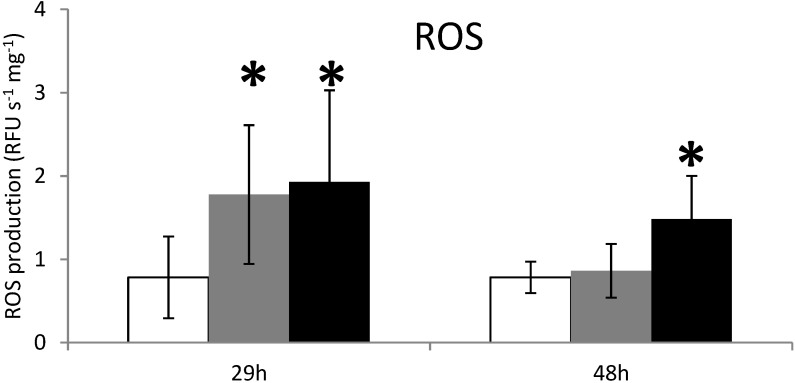
ROS production measurements in the digestive gland tissues of oysters not exposed (**white**) or exposed 29 h and 48 h to *Alexandrium catenella* (**black**) or to *Alexandrium tamarense* (**grey**). * *p <* 0.01.

Results show a significant increase (*p <* 0.01) in the production of reactive oxygen species (ROS) in DG of oysters at 29 h exposure to both non-toxic *A. tamarense* and toxic *A. catenella* (Figure 2). ROS production is not related to PSTs accumulated in DG at least for this exposure time. However, in the same experiment at a 48-h exposure time, ROS production remains high in oysters exposed to the toxic *A. catenella* (Figure 2). Knowing that the two *Alexandrium* species have nearly the same morphology and organic compounds, these results provide the first evidence that the presence of PSTs in the oyster DG induces directly or indirectly a permanent stress response in this organ after two days exposure to the toxic algae. Garcia-Lagunas [17] showed an increase of ROS production in *C. gigas* fed the toxic dinoflagellate *Gymnodinium catenatum*. In contrast, ROS production decreased when *C. gigas* hemocytes were exposed to the PSTs producer *Alexandrium minutum* [40], but was not modulated in hemocytes of *Crassostrea virginicas* exposed to the PSTs producer *Alexandrium fundyense* [19]. This suggests that specific PST compounds are related to ROS production. 

Multiple enzyme systems can contribute to the generation of ROS, but it appears that CYP450 family may have an important role in any case [41,42]. In the endoplasmic reticulum, ROS are produced by the microsomal monooxygenase system composed of CYP450 members, NADPH-P450 reductase (NPR) and phospholipids [43]. In mammals, the over-expression of *CYP1A* is associated with the production of ROS, including superoxide anion and hydrogen peroxide (H_2_O_2_) [44]. Even so, it has been difficult to clearly define the role of P450-generated oxidative stress in this experiment, but the production of ROS at 48 h of exposure could be generated by CYP1A activity. 

### 2.3. Apoptosis in Digestive Gland

**Figure 3:**
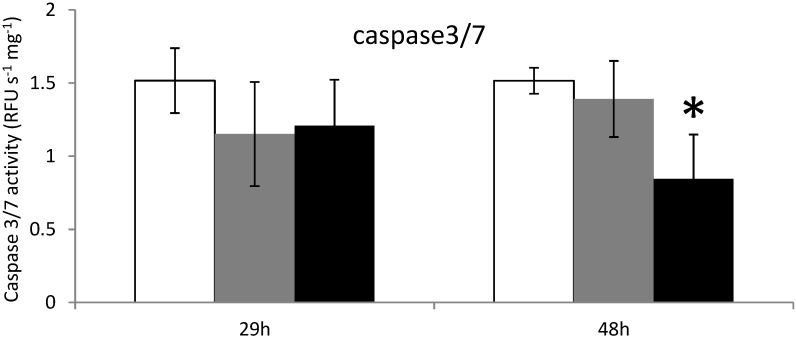
Caspases 3/7 activity measurements in the digestive gland tissues of oysters not exposed (**white**) or exposed 29 h and 48 h to *Alexandrium catenella* (**black**) or to *Alexandrium tamarense* (**grey**). * *p <* 0.01.

When *C. gigas* were exposed 29 h to the microalgae *A. catenella* or *A. tamarense*, no modulation of the caspase-3/7 activity was detected (Figure 3). However, after 48 h of exposure, caspase-3/7 activity decreased significantly in oysters exposed to *A. catenella*, but not in unfed oysters or oysters exposed to *A. tamarense* (Figure 3). 

**Figure 4:**
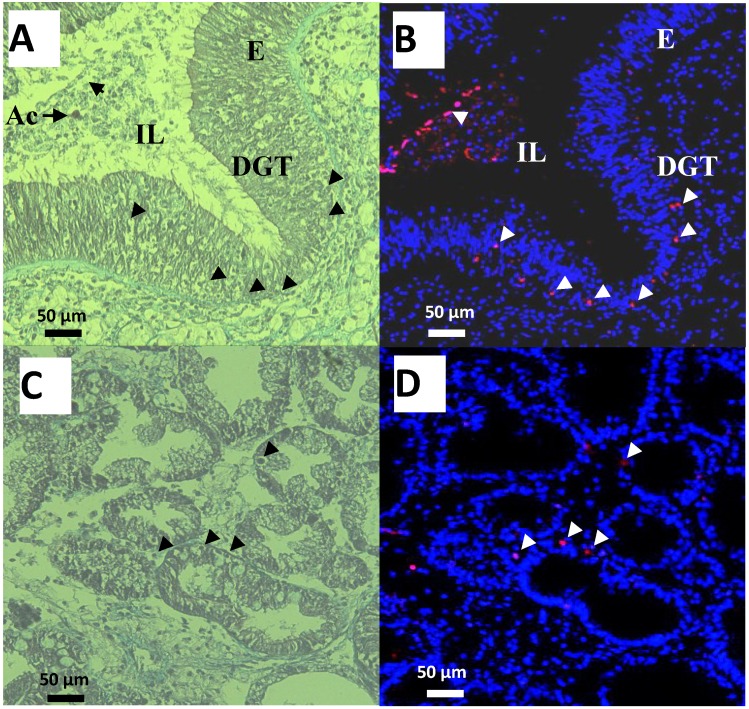
Observations of cells in the digestive gland tubules (DGT), intestine epithelium (E) and Lumen (IL) of oysters exposed for 48 h to *Alexandrium catenella* (Ac). (**A**,**C**) Trichrome de Masson staining (**B**,**D**) Terminal deoxynucleotidyl TransferaseTetra MethylRhodamine Nick End Labeling (TTMRNEL) staining (nuclei are stained in blue, nuclei of apoptotic cells in red (Δ)).

In addition, intact *A. catenella* cells were observed in intestine lumen (IL) of oysters exposed for 48 h to this microalga (Figure 4A). Thickness of digestive gland tubules (DGT) showed a dominant star-shape in the intestine lumen formed by digestive cells, indicating that the oysters were actively feeding and absorbing (Figure 4C). Moreover, no microalgae cells were observed in digestive tubules (Figure 4C). Epifluorescent observations of the corresponding sections showed few apoptotic cells through epithelia of the intestine (Figure 4B), intestine lumen (Figure 4B), and digestive tubules (Figure 4D). Similar results were observed in the DG of unfed oysters or oysters exposed to the non-toxic *A. tamarense* (data not shown). 

**Figure 5:**
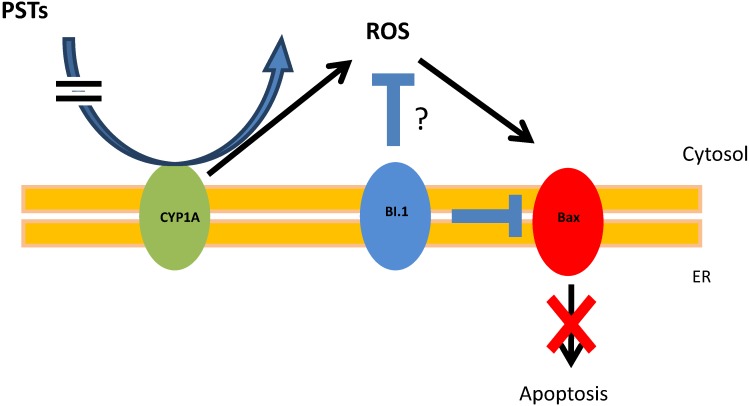
BI.1 regulates apoptosis induce by PST accumulation. In oyster stress cells, CYP1A can directly or indirectly neutralize and convert PST to ROS. ROS induces the expression of the protein Bax, which promote the intrinsic apoptotic pathway. BI-1 can inhibit Bax and could limit CYP1A production of ROS preventing the apoptosis.

In similar experiment, we previously demonstrated that exposure of oysters to toxic *A. catenella* induces apoptosis of circulating hemocytes even though Bax and executioner caspases were over-expressed before apoptosis occurred [20]. Interestingly, here we found no modulation of the mRNA expression levels of caspase-3/7 (Figure 1). Moreover caspase-3/7 activities decreased (Figure 3) and no significant apoptotic cells in tubules and in intestine were observed (Figure 4). A feedback mechanism for regulation of cells death process may occur. On the four anti-apoptotic molecules *Bcl2, BI.1*, *IAP1, IAP7B* tested, *BI*.*1* was significantly up-regulated at 48 h exposure. In mammals, several lines of evidence suggest that *BI*.*1* modulates the intrinsic apoptotic pathways [45,46,47] and inhibits ROS accumulation [48,49]. Similarly in the oyster, *BI*.*1* could play a key role for the control of ROS production and apoptosis by regulating Bax-induced cell death throughout the suggested mechanism presented in Figure 5. Apoptosis has been recognized to be an essential defense mechanism in oysters against invasion of pathogens [50]. Further studies should investigate whether the control of apoptosis in the DG related to PST contamination may promote the microbial pathogens (e.g., *Vibrio* sp.) invasion and, thereby, increase the susceptibility of oysters to disease.

### 2.4. PSTs Accumulation and Correlations with Natural Exposure to Alexandrium catenella

**Figure 6:**
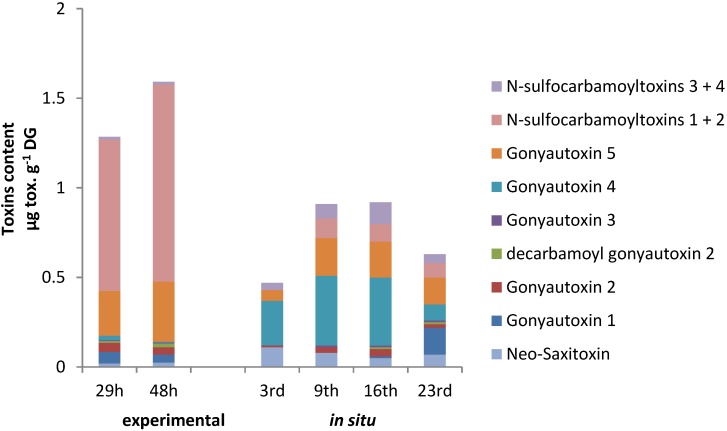
Evolution of the paralytic shellfish toxin (PST) content (μg/g DG wet weight) in the digestive gland of *Crassostrea gigas* individuals experimentally exposed to *Alexandrium catenella* (**left**) and to the natural toxic event occurring in may 2011 in Thau lagoon (**right**). The bar charts represent (in %) the temporal toxin.

The toxin content of *Alexandrium catenella* (ACT03 strain) was 5.3 ± 0.4 pg toxins/cell. The following toxins were found in decreasing proportion: *N*-sulfocarbamoyltoxins 1 + 2 (50.9%), Gonyautoxins 5 (35%), Gonyautoxins 4 (12%), Gonyautoxins 1 (1%) and neo-Saxitoxin (1%) with *N*-sulfocarbamoyltoxins 3 + 4, Gonyautoxins 3, Saxitoxin and decarbamoyl-Saxitoxin present as trace amounts (not shown). No PSTs were detected in *Alexandrium tamarense* (ATT07) strain. During the 48-h experiment, PSTs accumulated in the digestive gland of oysters fed *A. catenella* (Figure 6). 

The toxicity level reached 1.26 (0.17) and 1.56 (0.18) μg/g DG wet weight (μg Saxitoxin diHCl equivalent/g DG wet weight) at 29 and 48 h, respectively. The following toxins at 48 h were found in decreasing proportion: *N*-sulfocarbamoyltoxins 1 + 2 (69.1%), Gonyautoxin 5 (21%), Gonyautoxin 1 (2.8%), Gonyautoxin 2 (2.5%), decarbamoyl-Gonyautoxin 2 (1.4%), neo-Saxitoxin (1.6%) and *N*-sulfocarbamoyltoxins 3 + 4 (0.9%), with Gonyautoxins 3 and 4 present as trace amounts. This profile differs from *A. catenella* (ACT03) by an increase of the proportion of *N*-sulfocarbamoyltoxins 1 + 2 and a decrease in the proportion of Gonyautoxins 4 and 5 indicating that biotransformation occurred [51]. The rate of accumulation of PST in the tissues of marine bivalves is influenced not only by their feeding behavior (toxins intake rate), but also by the different chemical processes that take place in the bivalve tissues. Changes between the PST profile of *A. catenella* and the profile observed in DG of oysters may arise from epimerization of the accumulated toxins, chemical or enzymatic transformations and/or selective retention of the individual toxins [52,53,54]. 

**Figure 7:**
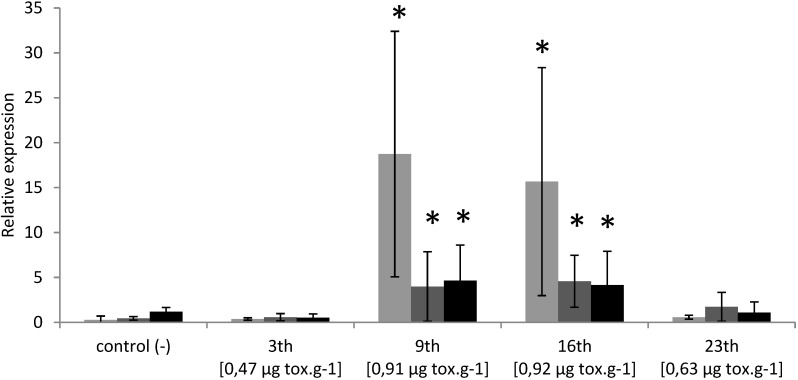
Relative expression of *cg-CYP1A* (**grey**), *cg-BI*.*1* (**dark-grey**) and *cg-Bax* (**black**) transcripts (normalized to ribosomal protein F40) in the digestive gland of *Crassostrea gigas* exposed to a PST event occurring in May 2011 in Thau lagoon. Bars represent mean ± standard errors (*N =* 5 animals).* *p <* 0.01.

PSTs accumulation in oysters exposed to toxic event in Thau lagoon reached 0.47 (0.26), 0.91 (0.34), 0.92 (0.33) and 0.63 (0.28) μg/g DG wet weight (μg Saxitoxin diHCl equivalent/g DG wet weight) on 3, 9, 16 and 23 May, 2011, respectively (Figure 6). PST profiles were characterized by the dominance of the Gonyautoxins 4 (14% to 53%) and low concentration of *N*-sulfocarbamoyltoxins 1 + 2 (0% to 12%). Similarly to experimental exposure *BI*.*1*, *Bax* and *CYP1A* transcripts levels were significantly over-expressed in DG of wild oysters collected on 9 and 16 May 2011, corresponding to toxin concentration 0.91 μg·g^−1^ and 0.92 μg·g^−1^ respectively (Figure 7). However, no modulation was detected in oysters collected on 3 and 23 May 2011, which corresponds to PSTs concentration of 0.47 μg·g^−1^ and 0.63 μg·g^−1^, respectively (Figure 7).

**Figure 8:**
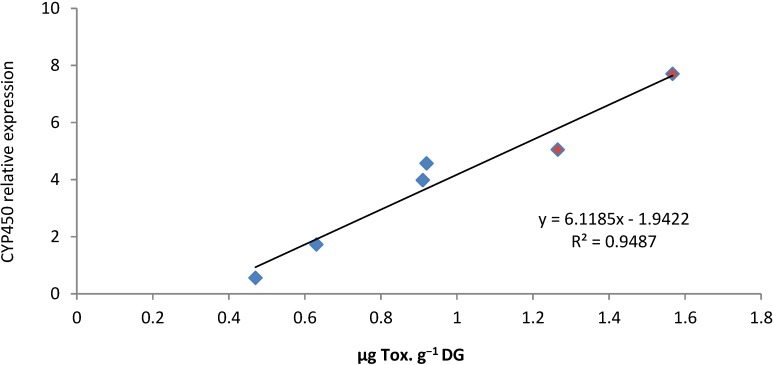
Correlation between the expression of *cg-CYP1A* transcripts (normalized to ribosomal protein F40) and PST concentration in digestive gland of *Crassostrea gigas* experimentally exposed (**red**) and *in situ* exposed (**blue**).

*CYP1A* have been described as a microsomal monooxygenase catalyzing the first step in biotransformation of many organic xenobiotics such as Polycylic Aromatic Hydrocarbon (PAH), Polychlorinated Biphenyl (PCB), dioxins, acrylamines, nitroaromatics [55]. Moreover, *CYP1A* regulation appears to be conserved in divergent groups of vertebrates and the elevation of CYP1A protein levels was demonstrated to be preceded by an increase in *CYP1A* mRNA levels following PAH exposure [56]. Our results show that *CYP1A* was up-regulated in DG of oysters exposed to *A. catenella*. Moreover, modulation of mRNA expression was demonstrated to depend directly on PSTs concentration (Figure 8). 

Such positive correlations have been found in feral fish between organic xenobiotics concentration and *CYP1A* expression level [57]. Although PSTs biotransformation associated to CYP1A activity has not been described yet, it could explain the difference between PST profile of *A. catenella* and that observed in DG of oyster exposed to *A*. *catenella*. 

Presently the mouse bioassay (MBA) still forms the basis of most shellfish toxicity monitoring programs. The procedure was developed more than half a century ago and has been refined and standardized by the Association of Official Analytical Chemists [58]. However, MBA holds a number of recognized disadvantages such as low sensitivity with a detection limit of 40 μg STX/100 g of shellfish tissue with a precision of ±15–20 percent. This test suffers from high variability, non-specific response, and the procedure raises ethical issues due to the use of laboratory animals. There are growing concerns about the continued use of mammals for bioassay. Alternative methods are developed such as chemical analyses using high performance liquid chromatography coupled to fluorescence detection (HPLC–FLD) and liquid chromatography/mass spectrometry (LC/MS) which are more specific and sensitive (detection limit of 3 μg/g STXeq 100 g^−1^) [59]. However, these methods require a high maintenance cost and skilled operators. Alternatively, immunoassay and open-sandwich immunoassay (OS-IA) methods are more sensitive than MBA and less expensive, but detect only specific PSTs of the 24 Saxitoxin (STX)-like identified [60,61]. When considered individually, the expression of genes related to detoxification (CYP1A) and apoptosis (Bax and BI-1) was not specific to the presence of PSTs in *C. gigas*. However, when these genes are considered together and knowing their potential role in the control of stress in oysters, their expression level could be a valuable biomarker to detect the stress induced by accumulated PSTs. Nevertheless, the use of CYP1A, BI.1 and Bax genes expression as accurate biomarkers requires further investigation because chemical and biological pollutants present in certain anthropized marine ecosystems could potentially modulate the expression of these genes. 

## 3. Experimental Section

### 3.1. In Situ Contamination

Wild adult Pacific oysters *Crassostrea gigas* individuals (≥60 mm, shell length) were collected from natural beds in Thau lagoon (N 43°26.058′ and E 003°39.878′, Bouzigues, Languedoc-Roussillon, France) in May 2011 when PSTs contamination of oysters was observed. Digestive gland tissues from 5 oysters were randomly taken at each sampling date, during 3rd, 9th, 16th and 23rd days of the month to perform genes expression and toxin analyses. 

### 3.2. Experimental Exposure to the Neurotoxic Alexandrium catenella

#### 3.2.1. Oysters and Microalgae

Adult Pacific oyster *C. gigas* were collected in November 2011 from an oyster farm in the Thau lagoon (Masson SARL, Languedoc-Roussillon, France) during periods when toxic events did not occur. The average total oyster fresh weight was 13.0 ± 2.9 g (average ± SD), average digestive gland weight was 1.8 ± 0.5 g and average shell length was 11.0 ± 1.0 cm. Before the experiments, oysters were exposed to a continuous flow of filtered (10 μm) Mediterranean seawater maintained in partial starvation, having only bacteria and nanoplankton to feed, at a constant temperature of 20 ± 1 °C during two weeks for acclimatization. 

The experiments were carried out with a toxic *A. catenella* (ACT03 strain) and a non-producer of PSTs, *A. tamarense (*ATT07 strain), isolated from the Thau lagoon in 2003 and 2007, respectively. The enriched natural sea water [62] culture medium was used to cultivate these two species and was characterized by a salinity of 35 PSU. The two dinoflagellates were grown in batch cultures at 20 ± 1 °C, under a cool-white fluorescent illumination (100 μM photons/m^2^/s) and a 12 h:12 h light:dark cycle. For the feeding experiments, we used algae in their exponential growth phase.

#### 3.2.2. Experiments

Two independent experimental exposures were carried out. For each experiment, after two weeks of acclimatization, 180 oysters were randomly placed into six tanks (30 individuals per tank) containing 10 L of filtered (0.2 μm) seawater. The experiments were conducted at a constant temperature of 20 ± 1 °C. Cells of *A. catenella* (two tanks) or *A. tamarense* (two tanks) were added into tank water regularly to maintain cell concentration (≥1 × 10^6^ cells/L) equivalent to that observed during a natural bloom in Thau lagoon [2]. In two control tanks, oysters were incubated in filtered (0.2 μm) sea water without microalgae. The mean concentrations in the tank water of the experiments for toxic *A. catenella* and non-toxic *A. tamarense* were (1.35 ± 0.02) × 10^6^ cells/L and (2.20 ± 0.23) × 10^6^ cells/L, respectively. Fresh cells were regularly added at 3, 6, 21 and 29 h to approach the initial cell concentrations. However, during the 48-h experiment, concentrations in tank water ranged between 1 × 10^6^ cells/L and 2.5 × 10^6^ cells/L. To estimate the concentration of cells in tanks during the experiment, triplicates of 1 mL of water were collected, and cells were fixed with Formalin (2%), and then counted in a Nageotte counting chamber using a photonic microscope (Cardinal Health, Dublin, Ireland).

#### 3.2.3. Tissue Sampling

Digestive gland tissues from 5 oysters randomly removed from tanks (containing 30 individuals each) were collected at time 0 (control), 29 and 48 h for gene expression analysis, apoptotic cells detection, caspase-3/7 activity and ROS production measurements. The remaining digestive gland were pooled and stored at −20 °C until the toxin extraction was performed. Hemocytes from oysters were also randomly taken from tanks at time 0 (control), 3, 6, 21, 29 and 48 h.

### 3.3. Genes Expression Analysis

The expression level of putative detoxification and putative apoptotic-related genes was measured in *C. gigas* exposed to toxic *A. catenella* or to the non-toxic *A. tamarense* at 29 and 48 h after the beginning of the experiment. In order to quantify the relative transcript levels of the selected detoxification-relative genes (*CYP1A*, *TransG*, *STrans1C1*, *GST and MT4*) and the apoptotic-related genes (*Bax*, *BI-1*, *Bcl2*, *caspase-3* and *caspase*-7, *IAP1* and *IAP-7B* and *FADD)*, digestive gland tissues were collected and placed in 0.5 mL of Trizol buffer and conserved at −20 °C. Total RNA was isolated from the oyster digestive gland using the standard Trizol method (Invitrogen Life Technologies SAS, Saint Aubin, France), then treated with DNAse (Invitrogen) to eliminate the contamination of genomic DNA. After sodium acetate precipitation, the quantity and quality of total RNA was determined using a NanoDrop spectrophotometer (NanoDrop Technologies, Wilmington, DE, USA) and agarose gel electrophoresis, respectively. Following heat denaturing (70 °C for 5 min), reverse transcription was performed using 1 μg of DG RNA prepared with 50 ng/μL oligo-(dT) 12 mer–18 mer in a 20-μL reaction volume containing 1 mM dNTPs, 1 unit/μL of RNAseOUT and 200 units/μL Moloney Murine Leukemia Virus Reverse Transcriptase (M-MLV RT) in reverse transcriptase buffer, according to the manufacturer’s instructions (Invitrogen Life Technologies SAS, Saint Aubin, France). 

The primer pairs used to quantify the expression level of apoptotic-related genes were designed according to the sequence available in GenBank. The expression of the ribosomal protein, F40, was used as the housekeeping gene control. All sequences of primers used for the amplification of apoptotic-related genes were published by Medhioub [20]. Sequences of primers used for the amplification of putative detoxification related genes are shown in Table 1. 

**Table 1:** Primers sequences for amplification, and size of the obtained products.

Gene	Primers Sequences 5′→3′	Product Size (bp)	GenBank ID
*Cg-CYP1A*	GACCGGAATCCAAGACTC GCAGTGTTTCCATGACGAC	70	CB 617404
Cg-TransG	AATCTACATCAAGAGTGACG AGGCTGCATTTCGTAAGAGG	144	EE 677859
Cg-STrans1C1	GGATGTCGAGTGTTCAGATG TTCTGTAGTGCTGGACTTTAG	119	CB 617550
Cg-GSTO	CTGTCCGTATGCTCAACGAG CTGCTGTGACTATTTGGACC	188	CB 617512
Cg-MT4	CAGCTCACACAGTCCCTTC CATGTACAGTTACACGATGC	143	AM 265551

Real-time PCR amplifications were performed in the Light Cycler 480 (Roche Diagnostics GmbH, Mannheim, Germany). In short, the following components were mixed to the indicated end-concentration: 5 mM MgCl_2_, 0.5 μM of each primer, 2.5 μL of reaction mix (Light Cycler^®^ 480 SYBR^®^ Green I Master mix, Roche Diagnostics GmbH, Mannheim, Germany) in a final volume of 5 μL. Reverse transcribed RNA (1 μL) diluted 1/10 was added as the PCR template to the Light-Cycler master mix, and the following run protocol was used: initial denaturing at 95 °C for 5 min; 95 °C for 10 s; 10 s at 58 °C; 72 °C for 10 s with a single fluorescence measurement; a melting curve program (65–97 °C) with a heating rate of 0.11 °C/s; a continuous fluorescence measurement; and a cooling step to 40 °C. Each PCR was performed in triplicate. To determine the qPCR efficiency of each primer pair used, standard curves were generated using six serial dilutions (1:1, 1:3, 1:7, 1:15, 1:31, 1:63) of a unique cDNA sample constituted from a pool of all cDNAs obtained from each condition; qPCR efficiencies of the tested genes varied between 1.85 and 1.99. Moreover, the real-time PCR product analysis on agarose gel and by melting curve revealed a unique lane and a unique peak, respectively, indicating the formation of a single PCR product with no artefacts (data not shown). For further expression level analysis, the Crossing Points (CP) were determined for each transcript using the Light Cycler software (Roche Diagnostics GmbH, Mannheim, Germany). The amount of apoptotic-related genes expressed was calculated relative to the amount of the ribosomal protein F40 housekeeping gene (because of its lower coefficient of variation) using the delta-delta threshold cycle (∆∆*Ct*) method [63].

### 3.4. Apoptotic Cells Detection Assay

A 5-mm cross-section including the digestive gland was taken from 10 oysters exposed for 48 h to the toxic dinoflagellates *A. catenella*. The dissected tissues were fixed in Davidson’s fixative [64] for 48 h. Cross sections were dehydrated in ascending ethanol solution (70°, 95° and 100°), cleared with LMR then embedded in paraffin. Then, 5 μm thick sections were cut, mounted on glass slides, paraffin was removed with LMR and tissues rehydrated in decreasing ethanol solution (70°, 50° and 25°) then wash with phosphate buffer saline (PBS). 

For each individual, two serial thick sections were performed from the DG cross section. One section was stained with Trichrome de Masson [65]. The DNA fragmentation that occurs at the early stages of apoptosis was detected by the red fluorescent label of DNA fragmentation (*In Situ* Cell Death Detection Kit, TetraMethylRhodamine (TMR) red, Roche Diagnostics GmbH, Mannheim, Germany) on the second section according to the manufacturer’s recommendations. Briefly, tissues were permeabilized for 8 min in PBS buffer containing 0.1% Triton X-100 and 0.1% sodium citrate. TMR red was added; then cells were incubated in the dark for 60 min at 37 °C. After three washes with PBS buffer, slides were cover with, 4′,6′-diamidino-2-phénylindole (DAPI) and incubated in the dark for 10 min. Upon staining, the fluorescent products generated by the two dyes can be visualized using a wide-field fluorescence microscope Olympus AX70 (Olympus corporation, Tokyo, Japan) equipped with standard red (540 nm–580 nm, TMRred) and blue (358 nm–461 nm, DAPI) filter sets.

### 3.5. Caspase-3/7 and ROS Activities Assays

An amount of 100 mg of digestive gland from 10 oysters (two tanks) non-exposed or exposed for 29 h and 48 h to the PST producer *A. catenella*, or the non toxic *A. tamarense* were crushed and homogenized in 0.5 mL of PBS buffer (Digestive gland suspension solution). The caspase-3/7 activity was performed by *the AFC SensoLyte Homogeneous AFC caspases-3/7 assay kit* (Anaspec). Briefly, 50 μL of the DG solution was mixed with 50 μL of caspases solution (50 μL caspases-3/7 substrate Ac-DEVD-AFC, 200 μL of DTT and 4.75 mL of assay buffer) then drop in black flat bottom 96-well plate. Upon caspases-3/7 cleavage, Ac-DEVD-AFC generates the AFC fluorophore which has bright blue fluorescence and can be detected at excitation/emission = 300 nm/500 nm. Fluorescence intensity of the samples is determined using the spectrometer TECAN^® ^(Tecan Group Ltd, Männedorf, Swiss). The kinetics of caspase-3/7 activity was determined in a continuous manner by recording the data every 5 min for 60 min. The ROS activity was performed by *Total ROS/superoxide detection Kit* (Enzo Life Sciences, Villeurbanne, France). Briefly, 50 μL of the DG suspension was mixed with 50 μL of 1 mM solution of 2′,7′-dichlorfluorescein-diacetate (DCFH-DA) then drop in black flat bottom 96-well plate. This molecule will penetrate through the cell membrane and the presence of intracellular ROS will cleave to release the fluorophore molecule DCF that can be detected by measuring the fluorescence at excitation/emission = 480 nm/530 nm. To assess specific activity of caspase-3/7 and ROS production, total proteins contents was determined by the BCA Protein assay (Bio-Rad, Marnes-la-Coquette, France) according to the manufacturer protocol. Samples were compared to a standard curve of bovine serum albumin (BSA). Results are expressed for ROS production and for caspases activities in relative fluorescence unit (RFU) per second per mg of protein. 

### 3.6. Chemical Analysis of PSPs by Liquid Chromatography/Fluorescence Detection (LC/FD)

The pooled digestive gland tissues were frozen at −20 °C until further processing. To extract the toxins, 5 mL of 0.1 N hydrochloric acid were added and the samples were mixed for 2 min with a high-speed homogenizer (15,000 rpm). The pH of the mixture was adjusted between 2.0 and 4.0 and the samples were centrifuged at 4200 g for 10 min at 4 °C [66]. The supernatants were filtered on 10 KDa PES filters and analyzed using the LC/FD PSP toxin analyses method of Van De Riet [59]. The toxins as GTXs, dc-GTXs, dc-STXs and STXs were separated by reverse chromatography using a RP column (Zorbax Bonus RP, 3.5 μM, 4.6 × 150 mm, Agilent Technologies, Massy, France) with a flow rate of 0.8 mL/min. The eluent pH and/or column temperature were optimize for the separation of some toxins (dc-GTX3/GTX5/dc-GTX-2 & C1/C2). The toxins were quantified using certified standards provided by CNRC (Halifax,Canada). An acid hydrolysis (HCl 0.4 N at 97 °C for 5min) was used to confirm the presence of GTX6 [67]. The C-toxins were separated by a Thermo Beta Basic 8 column (5 μm, 4.6 × 250 mm) with a flow rate of 0.8 mL/min. Triplicates of 10 mL batch cultures (cell concentration ≥ 10^7^cells/L) were sampled during the exponential growth phase of the cultivated dinoflagellates. After centrifugation (3000 g, 15 min, 4 °C), the cells were suspended in 1 mL of 0.1 N acetic acid and frozen at −20 °C. To release the toxins, the samples were sonicated for 60 min, and then centrifuged at 17,000 g for 10 min at 4 °C. The toxins analyses of filtered supernatants were performed as explained above.

### 3.7. Statistics

Data were analyzed using one-way ANOVA followed by Tukey test (Statistica 10.0 software, StatSoft, Maison-Alfort, France). Values are mean ± SD of 10 individuals from two independent experiments and 5 individuals from *in situ* sampling. * *p <* 0.01. 

## 4. Conclusions

This study is the first examine the relationships between PSTs accumulation and metabolic process that occur in the digestive gland of oysters exposed to toxic dinoflagellates. Results show that a feedback mechanism to control apoptosis and detoxification process occurs in the digestive gland of *Crassostrea gigas* experimentally exposed to the PSTs producer *Alexandrium catenella*. Moreover when considered together the expression level of genes implicated in those processes could be used as biomarker to assess stress caused by PSTs accumulation.
